# Pseudoaneurysm of the subclavian artery following clavicle fracture due to blunt traumatism: a case report

**DOI:** 10.11604/pamj.2020.36.262.22320

**Published:** 2020-08-11

**Authors:** Hicham Belmir, Anis Chagou, Youssef Tijani, Amine Azghari

**Affiliations:** 1 Department of Vascular Surgery, Mohammed VI University of Health Sciences, Casablanca, Morocco,; 2 Department of Traumatology and Orthopaedics, Mohammed VI University of Health Sciences, Casablanca, Morocco,; 3 Department of Vascular Surgery, Mohammed V University, Casablanca, Morocco

**Keywords:** Clavicle fractures, pseudoaneurysm, subclavian artery

## Abstract

The clavicle fractures are frequent, vascular injuries associated with closed fractures of clavicle are rare. The pseudoaneurysms of the subclavian artery constitute an exceptional complication. We report a case of a 40-year-old who presented an expanding hematoma of the right side of the neck after a road traffic accident. Radiography of the right shoulder showed a midclavicular fracture. An arterial doppler of vessels showed a circulating hematoma in the contact of the right subclavian artery with a correct distality flow. Computed tomographic angiogram of the chest confirmed the diagnosis of a false aneurysm in the postvertebral portion of the right subclavian artery. The treatment was surgical and consisted of excision of the false aneurysm and a repair of the arterial injury by an arterial patch, the clavicle was fixed with a reconstruction plate and screws. Early intervention appears to be indicated due to the risk of thrombo-embolic complications. Endovascular repair appears to be the preferred treatment modalities, due to a lower rate of cardiopulmonary complications, but it is reserved for much selected cases.

## Introduction

The clavicle fractures are frequent, they represent 2.6 to 5% of all the fractures [1]. The neurological hurts and especially vascular injuries associated with closed fractures of clavicle are rare [2]. The pseudoaneurysms of the subclavian artery constitute an exceptional complication. We report a case of a false aneurysm of the right subclavian artery in a 40-year-old patient after a road traffic accident. Through this observation, we are going to expose some diagnostic and especially therapeutic difficulties during this care.

## Patient and observation

**Figure 1:**
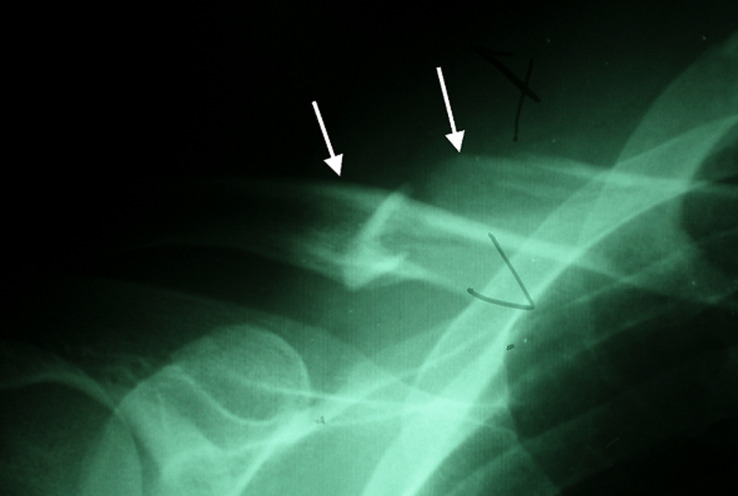
radiograph showing a right displaced clavicle fracture

**Figure 2:**
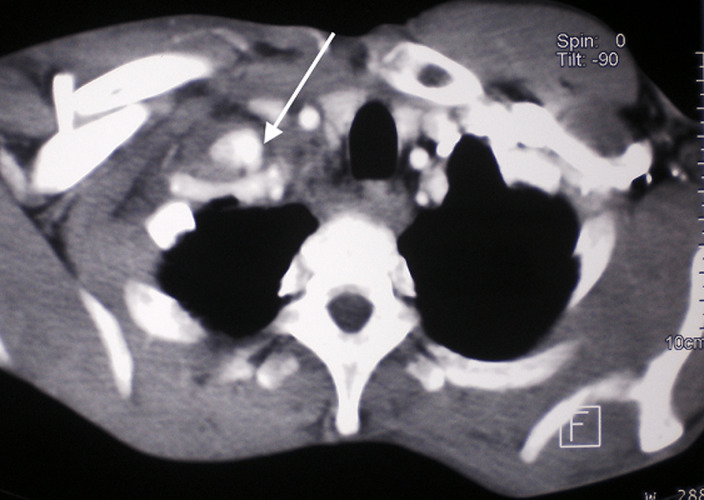
CT-angiography showing the pseudoaneurysm of right subclavian artery (arrow) in relation to the fracture

**Figure 3:**
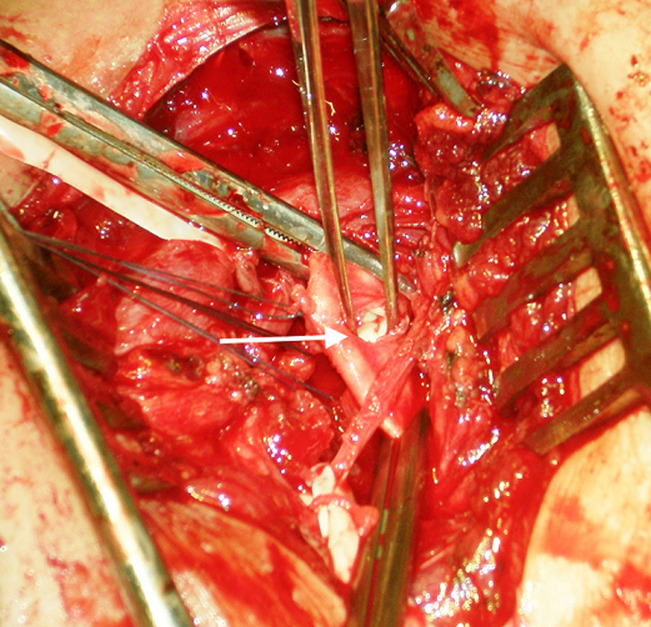
partial section of right subclavian artery due to the clavicle fracture with pseudoaneurysm neck (arrow)

**Figure 4:**
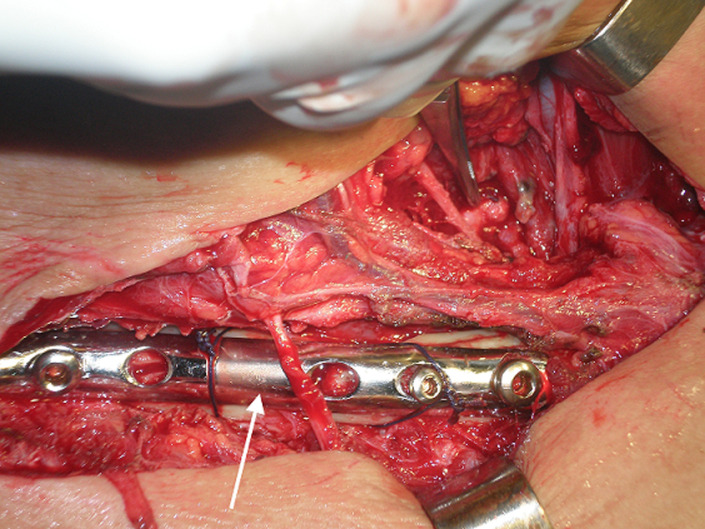
clavicular fixation with reconstruction plate after repair of the arterial injury

**Figure 5:**
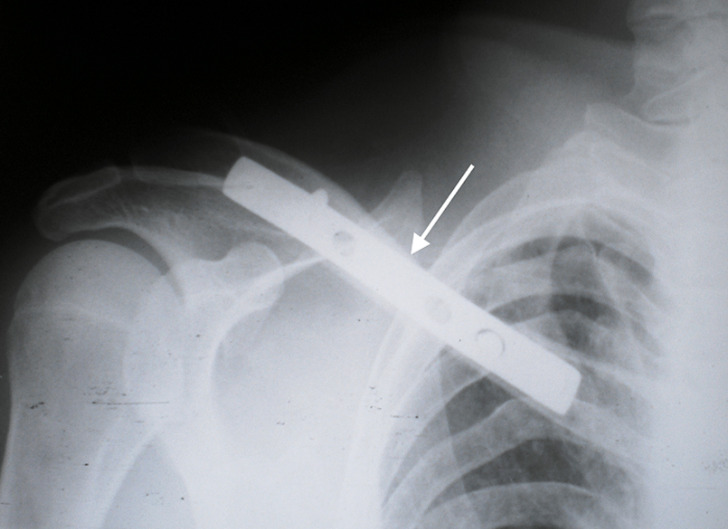
postoperative radiograph of clavicular fixation with reconstruction plate

A 42-year-old man was admitted to the emergency department for a right thoracic traumatism after a road traffic accident. The patient presented pain of the right shoulder with an oedema of the subclavicular region. He was hemodynamically stable. The physical examination found an expanding hematoma on the right side of the neck, there was no neurological deficit, ipsilateral radial and brachial pulses of the right upper limb were palpable. Radiography of the right shoulder showed a midclavicular fracture (Figure 1). An arterial doppler of vessels showed a circulating hematoma in the contact of the right subclavian artery measuring 4 cm in diameter with a correct distality flow. Computed tomographic angiogram (CT) of the chest confirmed the diagnosis of a false aneurysm measuring 4 cm in the postvertebral portion of the right subclavian artery (Figure 2). The hemogram showed a haemoglobin = 13g/dl. The patient was urgently taken to the operating room, a first vascular time was consisted of an excision of the pseudoaneurysm (Figure 3) and a repair of the arterial injury by an arterial patch taken from the right arterial trunk thyro-bicervico-scapular which was split, the second time was orthopaedic, the clavicle was fixed with a reconstruction plate and screws (Figure 4, Figure 5). The postoperative course was uneventful, and subsequent follow-up at 6 months revealed a well-perfused right upper limb.

## Discussion

To the best of authors´ knowledge, the incidence of subclavian vascular injury in closed fractures of clavicle has not been documented in the literature [3]. The incriminated mechanism is due to the direct consequences of the traumatism at the level of the top of the thorax, especially if a fracture of the clavicle is associated which would be responsible of an intimale hurt, even a partial section of the artery which would be responsible of the artery´s pseudoaneurysm [4]. Symptoms include an oedema, an expanding or pulsatile mass, weakness and paresthesias of the ipsilateral upper extremity, and ischemia in the upper extremity by migration of emboles. The arterial examination is necessary because the arterial injuries can be masked by the excellent collateral circulation surrounding the scapula [5]. In our patient, the physical examination revealed a pulsatile mass of the right subclavian region, the arterial and neurological examination was normal. Standard radiography shows a fracture of the clavicle, the humerus or the first ribs, and can show the existence of a pneumothorax or an associated hemothorax. Our patient had a right midclavicular fracture. The diagnosis of the pseudoaneurysm of the subclavian artery is confirmed on the arterial doppler, and on the computed tomographic (CT) angiogram of the chest which allows a completed lesion check-up, he also allows to measure the size and the diameter of the false aneurysm. The angiography can have a diagnostic and therapeutic purpose. In this observation, the diagnosis of the pseudoaneurysm of the subclavian artery was confirmed on the data of the CT angiogram. The therapeutic of a traumatic pseudoaneurysm of the subclavian artery can be surgical, or recently, many studies were interested in the endovascular repair and hybrid procedures, especially the use of cover stent-graft which are indicated if there is a difficult surgical access, and also if the patient is in a bad general condition [6-8]. The embolisation can be used if there is a narrow snare avoiding the risk of coils migration. Many complications can occur from the endovascular approach. The most commonly reported are access site hematoma or pseudoaneurysm, graft thrombosis, stenosis, cerebral embolization, and strut dislocation [9]. In our case, the fracture of the clavicle was much displaced with a shortening and an overlapping requiring a reduction and a fixation with a plate and screws, which needed a conventional surgical treatment of the false aneurysm.

## Conclusion

The false aneurysms of the subclavian artery after closed fracture of the clavicle are rare, early intervention appears to be indicated due to the risk of thrombo-embolic complications. Endovascular repair and hybrid procedures appear to be the preferred treatment modalities, due to a lower rate of cardiopulmonary complications, but it is reserved for much selected cases.
